# Nurses as the Pioneer New Women

**Published:** 1921-09-17

**Authors:** 


### THE MATRONS' AND SISTERS' DEPARTMENT.

### NURSES AS THE PIONEER NEW WOMEN.

To contrast the women of the present day with the women out of whom, under Florence Nightingale's leadership, they were evolved is to become aware that nurses have not only themselves taken the lead in embracing new ideals, but that with these ideals they have permeated the whole world of women by precept and by example. Florence Nightingale had probably little conception of the extent to which her new profession was destined to transform the women of the future. She endeavoured so to build up the nursing services that quiet reserved gentlewomen, hiding like herself from publicity, but braving it when necessary, might mould and direct the great company of submissive, strictly disciplined rank and file. The impress of this ideal of nursing rests on the nurse-training schools to the present day, but the type of woman which has emerged from them has become by degrees strongly differentiated from that which was anticipated. Nurses became the direct pioneers of the movement towards what may be called the woman of the future, for at the present moment the "new woman" is but half developed and comes short in a hundred particulars of what she might be.

It may fairly be claimed that it was Florence Nightingale and her disciples who gave the death blow to the theory that it was the part of all good women, more especially of the well-to-do, to sit at home, mind exclusively their own business, doubt their own powers and shrink from a knowledge of facts. To do otherwise was "unwomanly," and even Dickens, so acute in perceptions, could not resist the temptation to pander to the prevailing ideals of womanhood by bitterly satirising social endeavour in Mrs. Pardiggle and Mrs. Jellaby. Due mainly to the example of nurses the leaning habit, which glued a woman to her male relatives, a clog on them for life should she not marry, has gone for ever. Women who had learned their power to organise and rule in the wards could never again assume the attitude towards man of the ivy to the oak. They discovered one by one their own talents for direction and taught other women to do the like. They learned as they went along to believe in their ability to control their own environment and that of others. And the lesson has never been forgotten.

The great pioneer matrons of the Victorian age were evolved from shy, reserved women whose tastes were in favour of seclusion but who understood no recoil from the voice of duty. It was their glory that they broke through traditions which banned almost every natural impulse, and slurred the simplest domestic actions as vulgar and unseemly. These new women dared to lift the "menial" duties which all but the lowest had been taught to shun into a fine art, and demonstrated to women at large the saving truth that there is nothing in the realm of nature common or unclean. The bloom of womanhood, so lightly lost according to Victorian critics, rested visibly upon them, and the satirists for very shame held their tongues. The victory was won once for all, and even the cheery women scavengers of the war period may be hailed as the successors of those early nurses who knew no ward-maids and taught the world with their own hands the beauty of cleanliness.

Lastly, was it not nurses who taught women to hold back from no form of service, to dismiss fears and go wherever needed? From the very beginning nursing has been a propaganda. From the Nightingale Home and the handful of institutions where training could be obtained nurses went north, south, east, and west to bring tidings of a better way into one hospital after another, into Poor-law Infirmaries and workhouse sick wards, into private dwellings, into other lands and distant races. Nurses caught the inspiration one from another. No task was too heavy, no loneliness too appalling to deter volunteers from meeting each call as it occurred. The spirit of high endurance has always rested upon them as a corollary of their training, and from them other women have caught the fire. Long before the war tested alike the nursing profession and the laity, nurses had set their mark on dawning civilisation all over the world. They are helping at this moment to evolve new women of the right type in Africa, India, and other parts of the Far East. They have begun to be foremost in rescuing the women of the old, old degenerate type, and have ever laboured to bring into being the new mother.

Thus at length through several generations we arrive at the "New Nurse." Shall we concentrate attention on the silly habits some have learned to practice, and not rather look through to the spirit which animates by far the greater proportion of the newly certificated? We are teaching them more; we are training them better than ever before. The "new nurse," highly skilled, courageous, capable, full of zeal, is among the best types of the "new woman." She is self-confident because her strength lies not in self. She is successful in proportion as the mainspring of her actions is loving-kindness.

